# The Impact of Online Labor Platforms on Workforce Management in Health Care

**DOI:** 10.2196/68546

**Published:** 2025-05-02

**Authors:** Maryam Ahmadi Shad, Michael Simon, Florian Liberatore

**Affiliations:** 1 Department of Public Health, Institute of Nursing Science, University of Basel, Basel, Switzerland; 2 Winterthur Institute of Health Economics, Zurich University of Applied Sciences, Gertrudstrasse 8, Winterthur, 8400, Switzerland, 41 58 934 70 35, 41 58 935 70 35

**Keywords:** online labor platform, temporary work, workforce management, gig work, health care, personnel staffing and scheduling

## Abstract

Online labor platforms (OLPs) have the potential to change how the workforce is allocated and managed in health care. The contracting, coordination, and communication of bookings and work assignments happen on these platforms in near real-time with no delay and without any human interactions. This perspective paper describes the worldwide trend toward OLPs in health care, gives an overview of the functioning of these platforms, and discusses the prospects and challenges for health care management. As a real-world case, the platform logic, growth and traffic of a Swiss OLP designed for temporary nurse deployment are presented. OLPs facilitate managing different work arrangements (float pools and temporary work) through (1) offering health care staff flexible work options, which in turn lowers the dropout rates of health care professionals; and (2) effectively managing internal staffing allowing human resource sharing within and across health care organizations. For health care management research, OLPs yield data that can be used to analyze the characteristics, use, and dynamics of flexible work arrangements and temporary work in health care.

## Online Labor Platforms (OLPs): The Emerging Face of Temporary Employment in Health Care

For decades, health care organizations have relied on agencies to deploy temporary workers to fill their short-term vacancies and match workforce resources with care needs [1]. The use of temporary staff in hospitals is widespread globally. In the United States, about 75% of hospitals frequently employ temporary nurses [2]. Similarly, in the United Kingdom, the care hours provided by nonpermanent nurses increased more than doubled between 2012 and 2015 [3]. In Switzerland, though precise data are lacking, the use of temporary health care workers has risen since 2010. By 2022, temporary workers accounted for 3.5%-5.3% of health care staff, with agency nurses and care staff being the largest group [4]. With the emergence of OLPs, flexible work arrangements have undergone intense growth, and OLPs are increasingly replacing human match makers in temporary agencies for the intermediation between their temporary workers and their institutional clients.

In the United States, platforms such as Nomad, Medely, SnapCare, and CareRev have embraced the concept of temporary work by using web-based tools to connect hospitals with doctors and nurses for flexible, short-term assignments. Similarly, in Switzerland, web-based platforms like Careanesth, Coople, and Adecco serve as prominent avenues for the recruitment of temporary health care professionals.

**Figure 1.**
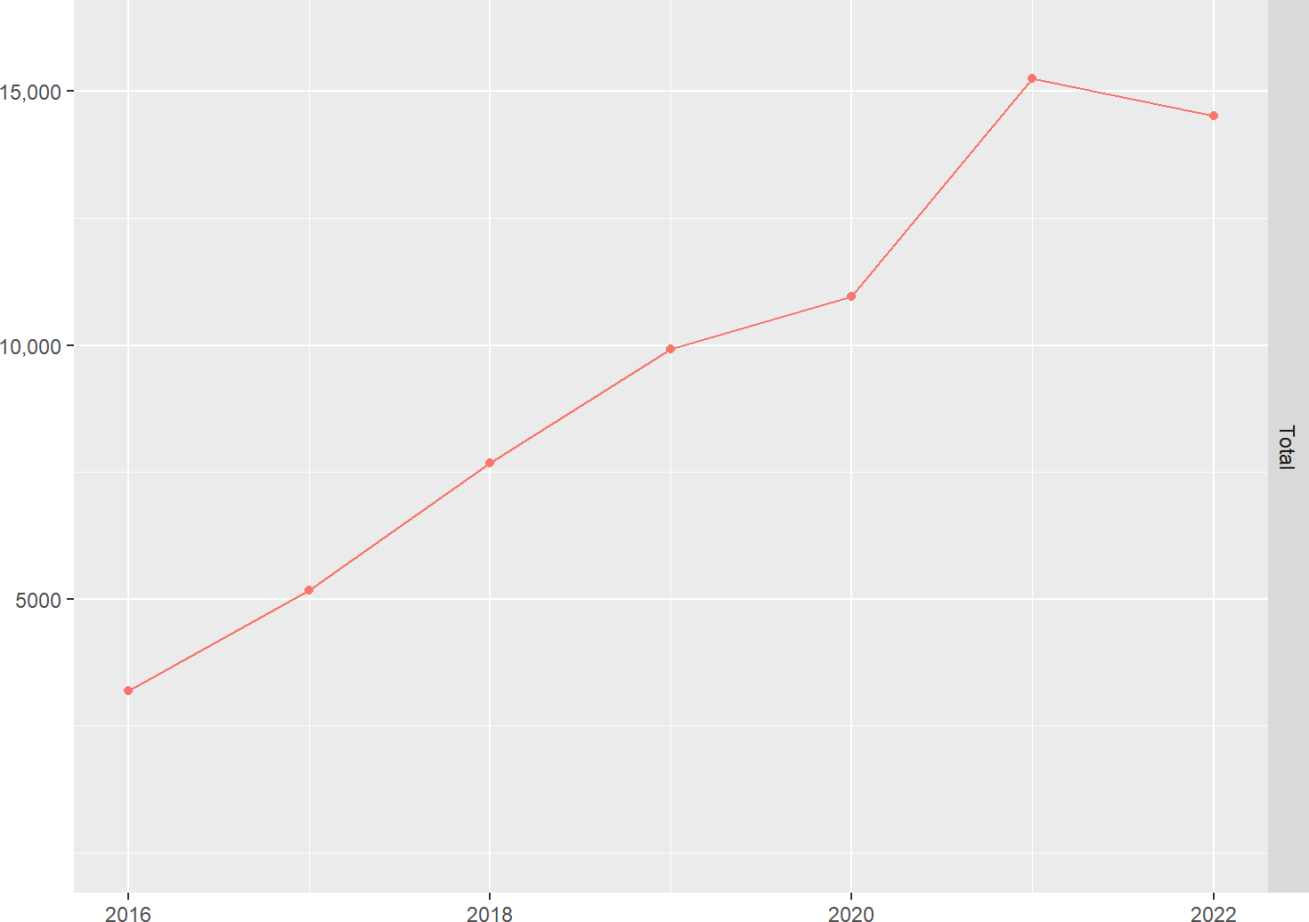
Growth of number of annual single shift bookings on a Swiss online labor platform.

Figure 1 shows the rising trend of annual shift bookings on Careanesth from 2016 to 2022. This trend has been largely neglected by health care managers as well as health services management research although OLPs could facilitate the efficient and effective use of temporary work and bring up new opportunities, challenges, and management issues for health care organizations with respect to processes and outcomes of recruitment, matching, administration, and evaluation of these arrangements.

OLPs leverage information and communication technology (ICT) to make a continuous, real-time matching of staff availability with the demands [5]. Individuals can accept or decline work contracts as they choose, without obligations. OLPs vary widely in functionality: some of them allow individuals to transact, while others match between organizations and individuals. Some of them support and facilitate web-based work and others coordinate onsite or offline tasks at client locations, including in health care settings [6]. In health care, OLPs may be implemented for internal health professional float pools in a single organization, float pools across organizations but also for the booking of external temporary staff.

While there are substantial literature exploring temporary work via OLPs in various sectors, there remains a notable limitation of evidence specific to health care. This gap is critical because the nature of platform work in health care diverges significantly from other industries as mentioned. The onsite provision of services introduces unique challenges and complexities that are not as common in other sectors. Moreover, in health care, platform workers are often highly qualified professionals like doctors and nurses, unlike the less specialized workforce found in other sectors. Managing this specialized workforce requires advanced strategies, including strategic shift planning, rapid response to fluctuating demands, and coordination of diverse teams comprising various health care professionals. These complexities underscore the need for robust and adaptive workforce management strategies tailored to the unique demands of the health care sector. The evidence around integrating ICTs into the recruitment of health care professionals shows advantages such as cost-effectiveness, wider reach, and time-saving compared to traditional methods of recruiting health care professionals [7]. Although ICTs could have positive effects, their implementation may also provoke concern. The growth of digital labor platforms presents regulatory challenges, particularly in cross-border contexts [8]. The systematic review by Gagnon et al [9] provides a broader perspective, emphasizing the impact of ICTs on workforce sustainability. They can support the recruitment and retention of health care professionals by improving job satisfaction, streamlining communication, and reducing administrative burdens. They have the potential to transform workforce management in health care, from operational scheduling to strategic workforce planning, addressing both immediate and long-term needs [9]. Despite its relevance, there is limited research on this topic and considering that this is a rising phenomenon, the existing evidence is relatively dated. Moreover, the existing studies primarily address the broader integration of ICTs into workforce management in health care, often overlooking the specific and unique application of OLPs for deploying temporary professionals. These studies predominantly focus on outcomes, leaving many aspects of the underlying dynamics of OLPs unexplored. Therefore, there is a critical need to understand the dynamics of OLPs and how the integration of them affects various aspects of workforce management. This includes evaluating the reliability of current data, identifying gaps, and understanding the potential benefits that can be derived from more comprehensive and accurate datasets.

Our perspectives in this paper describe the worldwide trend toward OLPs in health care, gives an overview of the functioning of these platforms, and provides an in-depth discussion on the impact of OLPs on health care workforce management, focusing on staffing practices, demand responsiveness, and team dynamics. By providing insights into these areas, we aim to help improve management practices and enhance the efficiency and effectiveness of health care delivery, ultimately leading to high-quality patient care.

### Ethical Considerations

The empirical results provided in this paper have been the subject of an ethical consideration by the Cantonal Ethics Committee of the Canton of Zurich within the Crowis project. The study does not fall under the Human Research Act and an exemption of an ethical review was received (BASEC-Nr. Req-2022-00708). The use of the per-diem nurse data of the platform was based on informed consent with an opt-out system. All employees of the temporary agency were informed by a letter of information about the study and could dissent the use of their individual data. The employee IDs as identifiers of the entries in the platform data were replaced by an alphanumeric code by the temporary agency to guarantee confidentiality. Platform data were stored in a server protected by a password and accessible only to the members of the research team. No compensation was paid to the temporary nurses for data provision.

### Operational Dynamics of OLPs and the Evolution of Workforce Management Practices

OLPs impact the whole process within the triangle of temporary agencies, temporary workers, and organizations booking services which can be divided into five steps: (1) enrollment in the platform; (2) offerings; (3) matching mechanisms; (4) administration; and (5) evaluation. The impact of OLPs on these steps is described in detail in the following.

#### Step 1: Enrollment

It typically refers to the process through which temporary workers register or sign up to become part of the platform’s pool of available temporary workers. The registration requires a health care professional’s basic personal information, professional credentials, and work experience. After the verification of the information and the certificate check by the platform, temporary workers may be required to review and sign agreements or contracts outlining the terms of their engagement with the platform, including payment terms, confidentiality agreements, and code of conduct. These profiles serve as a reference for health care facilities when they are looking to deploy temporary staff.

On the other side, organizations using OLPs need to provide key information to ensure smooth operations and compliance with platform standards. This includes clear details about shift offerings, payment terms such as hourly rates, payment methods, and timelines, along with guidance on administrative procedures. Additionally, organizations must share information about relevant labor laws and regulations that apply to health care workers, including their rights, protections, and available support mechanisms [6]. By transitioning from traditional agency-based enrollment methods to web-based platforms, temporary workers benefit from a more efficient, transparent, and accessible process for joining the health care workforce. It enhances communication and empowers health care professionals to manage and update their profiles effectively in real-time, reflecting changes in their professional status or preferences. Referring to the exemplary use case of the Swiss OLP, in 2022, 78 hospitals, 33 long-term facilities, 30 home-care organizations, and 11 other health care institutions were clients of the platform. On the platform, float pool as well as per-diem nurses who are employed by the platform provider can be booked for single shift assignments. A total of 283 per-diem nurses and 227 float-pool nurses were enrolled on the platform. Per-diem nurses can be booked from all institutions which are clients of the platform whereas float-pool nurses can be booked exclusively by each institution within their defined float pool, administered by the platform provider (Dettling M, Liberatore F. Unpublished data, April 2025).

#### Step 2: Offerings

**Figure 2.**
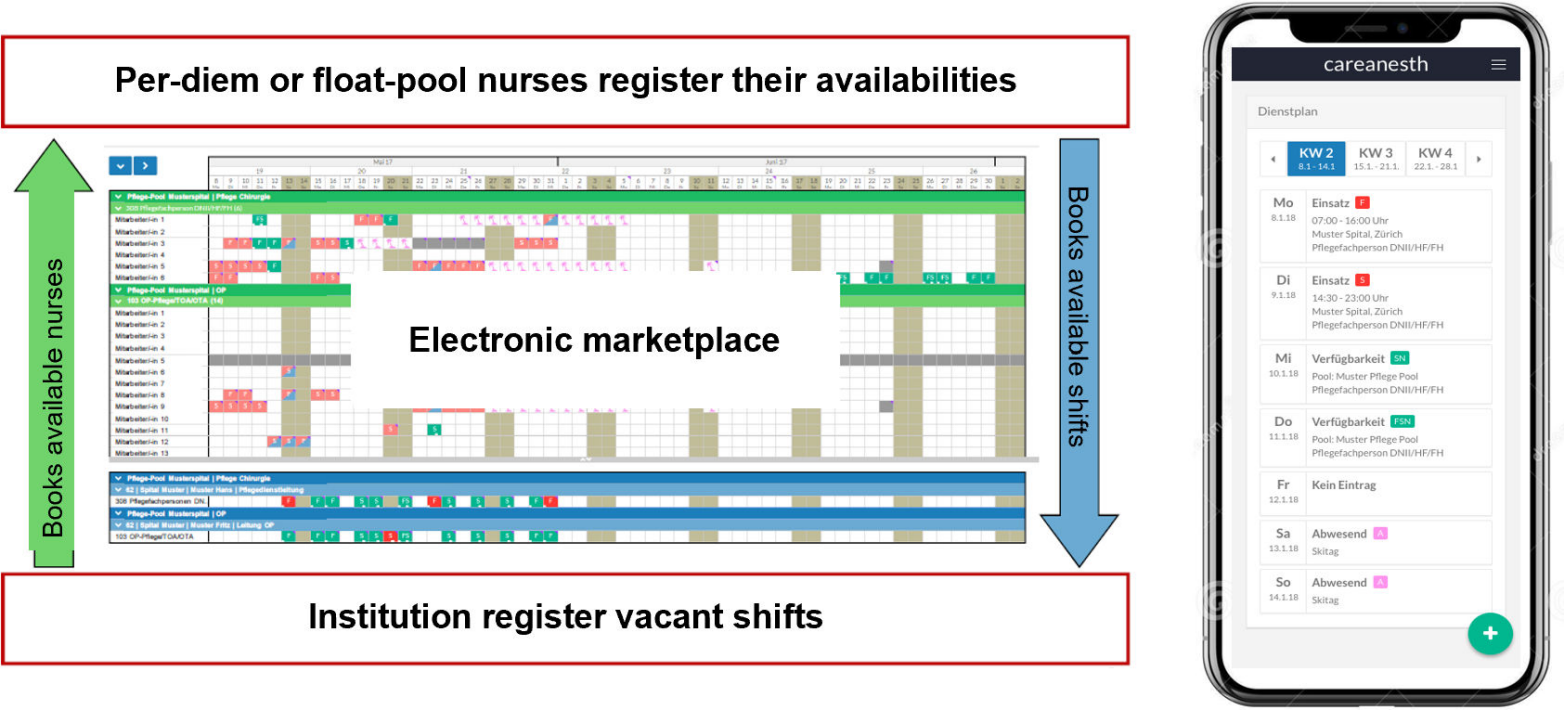
Managing shift offerings via the web browser or smartphone app.

**Figure 3.**
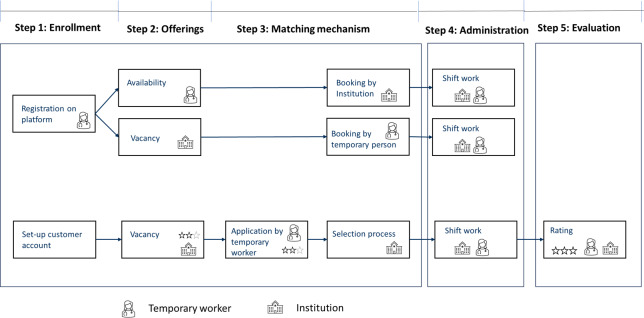
Overview about steps and general process on online labor platforms in health care settings.

Temporary workers and organizations can use a web browser or smartphone app to manage shift availability and vacancies (Figure 2). Health care professionals post the days and specific shifts they are available to work (eg, Monday, morning shift), while organizations post vacant shifts, specifying the ward type, required qualifications, and shift details (Figure 3).

Transitioning from the agencies to web-based platforms, health care professionals and hiring organizations have greater accountability and autonomy in leading transactions. Furthermore, OLPs provide the flexibility for hiring organizations to collect a group of “preferred” workers to prioritize them for job offerings. This differs from temporary agencies, where hiring organizations depend on human intermediaries in the agency to request temporary workers. This new model empowers both parties by eliminating the need for agency middlemen and streamlining the hiring process.

#### Step 3: Matching Process

A match on a platform can either result from an organization booking a shift availability or from a temporary worker taking on a vacant shift offer. Both lead to a binding agreement regarding the shift assignment. However, in some OLPs, the initiation process stems solely from the organization’s end, attracting a diverse pool of temporary job seekers. Here, applicants submit their applications, entering a selection process where the organization evaluates each individual against their criteria. This approach allows the organization to handpick the most suitable applicants. Matching mechanisms can follow the first come–first serve rule, which means that the first organization or temporary worker books the availability or vacant shift offer, and there is a binding agreement on the work. The exemplary case of the Swiss OLP uses this mechanism. In the booking process, the institutions see which type of nurse (registered nurse; vocational trained nurse assistant [Fachangestellte or Fachangestellter Gesundheit]; and intensive care nurse specialist [Intensivpflege]) is available for which shifts and have access to the individual curriculum vitae of the nurses to inform about qualifications and experience. Another possibility is that the organizations or temporary workers can apply for the offer and then after an evaluation by the contracting partner the contract is negotiated. (Figure 3). The web-based platform of Coople is an example that uses this mechanism. Alongside the bookings by hospitals and clients, some platforms use algorithms with and without artificial intelligence to analyze the data collected from both sides—organizations and professionals. These algorithms use various criteria and techniques to match the right health care worker with the right offer. Criteria may include skills, experience, location, availability, preferences, and historical performance data.

Platforms as managed service providers streamline the process of booking by allowing hospitals to access multiple temporary agencies through a single platform. This centralized approach simplifies staffing management and enhances efficiency for health care organizations. Hospitals can easily compare available temporary staff from various agencies, view their credentials, and make bookings according to their specific needs.

On OLPs, prebooking and prioritization rules are typically established to optimize the matching process between health care workers and organizations while ensuring efficient allocation of resources. These regulations may vary depending on the specific platform. These rules consider factors such as urgency of need (prioritize matches for immediate or time-sensitive vacant positions), availability (prioritize matches for only available workers), specialization, ratings, geographical proximity, repeated bookings in the past, premium services, and dynamic pricing (pricing algorithms may take into account factors such as demand, supply, and worker ratings to determine pricing for prebooked assignments, incentivizing workers to accept priority assignments) [7].

While OLPs primarily rely on ICT for matchmaking, traditional temporary agencies use “human matchmakers,” such as agency representatives, to connect workers with short-term job opportunities. However, this does not mean temporary agencies avoid using technology. They use ICT to collect and manage data on their contingent workforce, including qualifications, work experience, and current assignments. Human match makers then use this information to assign workers to clients. In contrast, OLPs function without human intermediaries, offering a web-based marketplace where labor supply and demand directly meet [5].

#### Step 4: Administration

The contracting, coordination and communication of a work assignment happens in near real-time with no delay. The platform usually requests a fee for the service from the platform provider like fees for a personnel agency for the placement service or licensing fees for the use of the platform as health care organizations.

When comparing the contracting processes between traditional temporary agencies and OLPs, distinctions emerge, especially in how they engage with temporary workers. There exist 2 primary models within OLPs: co-employment platforms and freelance platforms, each with their distinct characteristics. Co-employment platforms establish an employment relationship with their workers, subjecting them to collective labor agreements either within the platform firm or with client organizations. Consequently, these platforms typically offer a fixed hourly pay rate to their temporary workforce, with potential surcharges during periods of labor scarcity. Conversely, freelance platforms engage solo self-employed workers who operate as independent contractors, allowing them to set their own hourly rates. The negotiation of pay rates can vary, with either the freelance worker or the client organization proposing rates depending on the platform’s technical setup. At the same time, to avoid “races to the bottom,” some of the freelance platforms’ digital interfaces do not allow workers and hiring organizations to set hourly pay rates below a minimum level [5].

Additionally, automatic contracting as a key feature of OLPs facilitates the automatic generation and execution of contracts between the parties involved in a booking or transaction. This process is seamless and transparent, with the platform creating a standardized contract template based on the agreed-upon terms and relevant policies. These contracts typically outline key details such as service expectations, payment terms, duration, and other conditions. Once created, the contract is automatically sent to both parties for review and acceptance. This automated approach simplifies transactions, reduces administrative workload, and ensures clear communication and accountability for both parties.

In the use case of the Swiss OLP, the booking of a shift availability by an institution or the acceptance of a vacant shift by a float-pool nurse leads to a binding agreement between the institution and the temporary nurse. The matching (an assignment being offered and accepted) may occur as late as 24 hours before the start of the shift in question. The contracting, coordination and communication of a work assignment happens over the platform in near real-time with no delay.

#### Step 5: Evaluation

Some OLPs feature advanced ratings and reputation systems that foster trust and transparency between users. The Quality Rating 360 empowers both parties to provide comprehensive feedback on performance, behavior, and competencies, ensuring a fair and reliable marketplace. Temporary workers can rate organizations on factors like work environment, communication, and overall satisfaction, helping guide fellow professionals in making informed choices. Conversely, organizations can rate temporary workers based on professionalism, performance, and reliability, contributing to a dynamic feedback loop that enhances platform quality and accountability. Unlike traditional agencies, this feature grants the users valuable insights into the reputation and performance of various entities, enhancing transparency and informed decision-making. In the use case of the Swiss OLP, no rating system has been established to date but is planned.

## The Prospects and Challenges of OLPs for Health Care Management

### Impact of Using OLPs

The use of OLPs for temporary deployments has a significant impact on organizational management endeavors related to shift planning, the availability of new work arrangements, patient flow management, and the opportunities to share resources within and across organizations. However, the impact is associated not only with prospects but also with new challenges for health care managers.

### Managing Flexible Workforce in Shift Planning

In terms of workforce management, there are both benefits and challenges for hospitals whose workforce includes temporary staff. On the one hand, the web-based booking mechanisms, facilitate the staffing of shifts according to demand and make the covering of short-term absences more efficient (eg, by requiring fewer phone calls and emails) [8]. On the other hand, the operations on these platforms bring new administrative burdens for managers. They must draft and publish vacant shifts, and select temporary health care workers according to their shift demands, and availabilities of temporary staff [5]. Competencies and work experiences of temporary health care workers must be assessed based on the provided nurse-related information on the platform without personal contact or assessment [9]. Further, on OLPs, availabilities for unpopular shifts (eg, night shifts or shifts on public holidays) may be much less. Because OLPs act as a labor market, shift managers must adopt selective booking behaviors. This means that they do not book temporary workers for popular shifts and instead post vacancies for unpopular shifts, and temporary workers are forced to adapt their operational availability to the demands of the hospital in order to work there. Moreover, the monitoring of compliance with maximum working hours regulations and rest periods is challenging for health care institutions because the temporary health care workers may work in various institutions based on multiple work contracts with agencies and health care institutions in parallel.

### Offering New Work Arrangements for Health Care Professionals Using OLPs

The digital platform economy provides opportunities for new and diverse work arrangements. It potentially enables mixed contracts where individuals can engage in part-time, freelance, or fixed work simultaneously, reflecting the evolving nature of work in this digital era. The web-based booking systems save temporary workers from being permanently on call. Either they offer their shift availabilities which are most suitable to their individual preferences and obligations [10] or they consider overtaking vacant shifts offered by organizations that pop up on the platform. The decisions can be made without any obligations. This feature of self-scheduling in new work arrangements has been associated with higher job satisfaction and retention. However, nurse managers must cope with greater complexity due to the mixture of various temporary work arrangements in the shift planning. The lack of organizational boundaries in the work arrangements poses the challenge of assessing the competencies and experiences of temporary nurses without personal contact or assessment [9]. Health care managers must find solutions to overcome the resistance to change and install working contexts in which ad hoc teams with a mixture of permanent and temporary staff can work together in an efficient and effective way.

While for some health care professionals, these working models offer new opportunities, others may prefer traditional working and staffing models. Platform work increases the potential for worker exploitation and the workers have to deal with challenges caused by platform-based vulnerabilities such as inequality and precarity; which refers to the short-term contingent nature of the work and lack of clear training and career promotion [11,12]. As platform workers are most likely to be considered self-employed, rules on data protection and privacy become challenging. There is very little information on how platforms protect data and the privacy of workers as well as organizations. Concerns have also been raised that platforms possess an inequitable advantage over conventional agencies, as platforms have fewer responsibilities for working conditions such as issues related to the data privacy and intellectual property rights of temporary workers [6], making it challenging for health care institutions to comply with these regulations using temporary health care workers from these platforms. Furthermore, issues such as wage discrimination can occur on OLPs during the matching process due to varying wage structures. Some platforms let organizations set hourly rates, potentially leading to inconsistencies. Others offer fixed rates, possibly undervaluing experienced temporary workers. Platforms with flexible rates based on factors like experience may unintentionally perpetuate disparities. Additionally, fluctuating rates based on demand-supply dynamics could result in unequal pay.

### Sharing Resources Within and Across Organizations Using OLPs

OLPs are instrumental in promoting resource sharing within and across health care organizations. They facilitate sharing health care professionals across different organizations or units using 1 single tool and without the need for human matchmaking in each organization or unit. This approach aligns with the contemporary trend of using technology to bridge gaps and streamline interactions within and across organizations. Regarding sharing the workforce within and across organizations through OLPs, several challenges arise. Appropriate rules and mechanisms for prioritization, booking or prebooking, cost allocation, and activity tracking need to be defined to optimize resource use and enhance user experiences in the sharing economy paradigm. Prioritization in workforce management is essential for efficiently allocating resources and ensuring that the right people are in the appropriate roles at the correct time. Further, yield management and dynamic pricing approaches may be applied within the booking mechanisms of OLPs to foster the market mechanisms on these platforms resulting in a better allocation of the shared resources. Yield management as a dynamic pricing approach can be used to control capacities by adjusting prices based on real-time demand, supply, and market conditions. The sharing of health care professionals across organizations may be challenging because of obligatory minimum staffing regulations and its verification by nurse-patient ratios depending on the health care system setting.

### Effects of OLP Application on Patient Flow Management

Another significant advantage offered by OLPs is their facilitation of patient flow management and staff rescheduling within health care organizations. These platforms provide real-time access to data regarding the availability of health care professionals. This enables facilities to promptly address staffing gaps and adjust patient flow plans and staffing levels, accordingly, ensuring appropriate staffing levels in unexpected surges in demand [5]. Furthermore, the retrospective data capabilities of OLPs facilitate proactive workforce planning for both fixed and temporary staffing level requirements. By examining the trends and patterns, health care facilities can potentially forecast future demands and make strategic decisions about resource allocation to effectively address patient needs. This data-driven information enables organizations to align their staffing schedules more precisely with anticipated demand, thereby maximizing resource utilization and boosting the overall efficiency of patient flow management.

## Propositions for Future Research on Online Health Care Labor Platforms

OLPs and their application for the recruiting and management of temporary health care workers offer several propositions for future research.

First, studies should calculate and compare changes in administrative costs (cost savings vs new administrative burdens) using OLPs for the application of temporary work arrangements in health care organizations. Second, future research should address the impact of different offering and matchmaking rules on OLPs on matching rates and labor costs to understand better how OLPs allow health care organizations to apply and rely on dynamic shift planning systems with a mix of permanent and flexible staff. Third, the consequences of the increasing use of short-term single-shift assignments arranged via OLPs on treatment processes, costs, and outcomes require more research. Fourth, the market dynamics on OLPs and the leverage of health care organizations altering the supply side on these platforms is an important area informing health care managers how to behave optimally on these OLPs. Fifth, OLPs yield data that can be used to analyze the characteristics and dynamics of temporary work contributing to a greater understanding of their behavior as well as the use of temporary nurses in organizations. Compared to findings from surveys of temporary agency managers and temporary health care workers yielding motivations and behavioral intentions for temporary work, data from platforms represent actual behavior and therefore offer a sound basis for discussion on the extent and patterns of temporary work in a health care system. More specifically, platform data includes the following information.

- Data about the organizations and temporary health care workers operating on the platform inform about the sociodemographics of the temporary health care workers and organizational characteristics.
- Shift availabilities show how many and which shifts temporary health care workers offer thereby revealing their work preferences. Shift offers by the organizations on the platforms inform about the volume and characteristics of unstaffed shifts.
- Matching data displays how often temporary health care workers are booked, in how many different organizations they work, and in which types of shifts they are deployed. Further, matching rates provide information about the effectiveness of the platforms matching temporary health care workers’ availabilities and shift demands.

Overall, it can be concluded from the perspective paper, that health services management research should increase research activities in the field of OLPs in health care. The potential of allocating health care workers to their needs, but also according to the demands of the health care organization is a key topic for health care systems worldwide.

## Abbreviations

ICT: information and communication technology
OLP: online labor platform
